# Supplementary material for: Nst1, Densely Associated to P-Body in the Post-Exponential Phases of Saccharomyces cerevisiae, Shows an Intrinsic Potential of Producing Liquid-Like Condensates of P-Body Components in Cells
Source: Int J Mol Sci. 2022 Feb 24;23(5):2501. doi: 10.3390/ijms23052501 (PMC8910029; doi:10.3390/ijms23052501)
Supplement: Supplementary file 1 [file ijms-23-02501-s001.zip › ijms-1570866-supplementary.pdf]

International Journal of
Molecular Sciences

MDPI

Supplementary Materials

Nst1, Densely Associated to P-Body in the Post-Exponential Phases of *Saccharomyces cerevisiae,* Shows an Intrinsic Potential of Producing Liquid-Like Condensates of P-Body Components in Cells

Yoon-Jeong Choi and Kiwon Song *

Department of Biochemistry, College of Life Science and Biotechnology, Yonsei University, Seoul 03722, Korea; yoon3004@yonsei.ac.kr
* Correspondence: bc5012@yonsei.ac.kr; Tel: 82-2-2123-2705; Fax: 82-2-362-9897

Fig S1

A

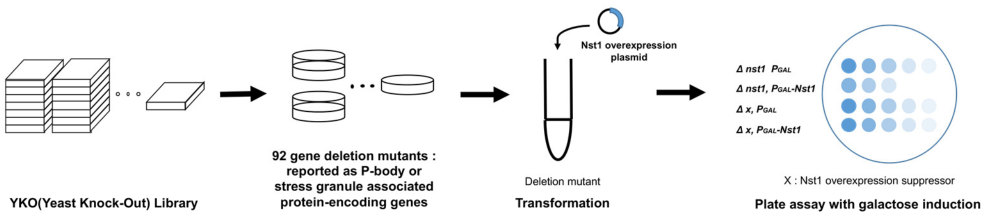

B

PB or SG associated genes in YKO library	Suppressors
RBG1,FUN12,CCR4,SSA1,HSP104,PSP2,CLU1,FAA4,DSS1, GIS2,CPA1,VTS1,MRN1,DBP1,SYH1,NGR1,YBR238C,LSM6, NPL3,EDC3,PRE9,PBP1,RPS0A,SBP1,SUI2,KSP1,LSM12,GBP2, SRO9,TIS11,SAM1,PAN3,TMA19,TEF4,DCS2,RPB4,RPN13, DCS1,EBS1,CAM1,RQC2,SCD6,TIF3,SEM1,YHB1,FUS3,HEK2, TIF4632,PAN2,WHI3,IGO1,TIF1,SSD1,IRC3,NOT3,YVH1,MDY2, LSM1,MET5,PAT1,PPH21,PUB1,HSP26,MKT1,RIE1,DED1, EAP1,CYS4,HCR1,SNO4,PBS2,POP2,PBY1,VMA2,SMY2, PBP4,PSP1,HSP31,ARC1,XRN1,KAP123,ECM32,BMH1, SAM2,SLF1,BLM10,SLH1,MAP1,DHH1,NRP1,NOT5,TIF4631	*PSP2, LSM12, DCS1, TIF4631, POP2*

C

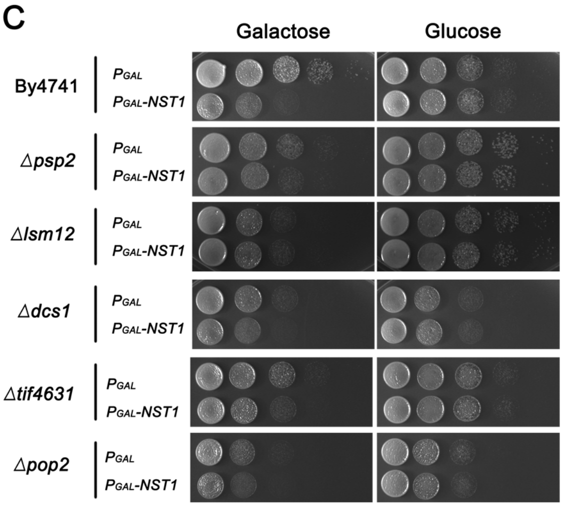

Figure S1. Genetic suppressor screening for the overexpressed Nst1 phenotype. **(A)** Schematic diagram of the genetic screening. Ninety-two gene deletion mutants related to P-bodies (PBs) or stress granules (SGs) were collected from the yeast knock-out (YKO) Library. Each strain was transformed with *pMW20-pGAL* and *pMW20-pGAL-NST1*. Transformed cells cultured in SC-U + 2% glucose were transferred to SC-U + 2% raffinose medium and then spotted on SC-U + 2% glucose and SC-U + 2% raffinose + 2% galactose agar plates. The spotting process is described in detail in the Section 4. **(B)** List of deletion mutants used in the genetic suppressor screening and the suppressors identified in the screening. **(C)** Spotting assay of the suppressors identified in the screening.

Fig S2

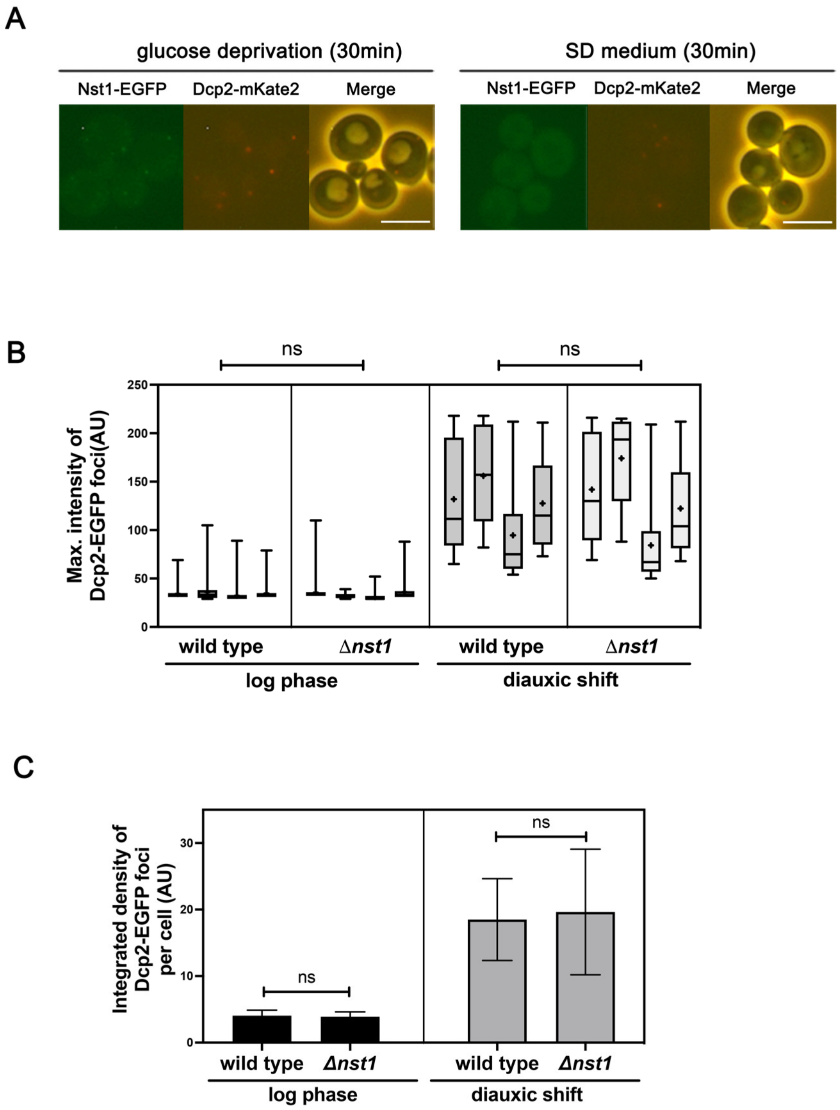

Figure S2. *NST1* deletion does not affect Dcp2-enhanced green fluorescent protein (EGFP) condensation in the diauxic shift. **(A)**Monitoring Dcp2-EGFP condensation in *nst1* deletion mutant. The *nst1* deletion mutant (YSK3506) and the wild-type (YSK3485) cells, whose chromosomal *DCP2* was tagged with *EGFP*, were primarily grown to exponential log phase and further cultured to the diauxic shift (OD_{600} 2–3). More than 150 cells of each strain were monitored in one independent experiment, and each experiment was repeated four times. Pixels with the highest 0.05% Dcp2-EGFP signal intensity were segmented for analysis. Segmentation and quantification methods applied to the images are described in Section 4. **(B)** Maximum signal intensities of Dcp2-EGFP puncta in the wild-type and *nst1* deletion mutants. The maximum intensity of the segmented puncta is plotted on the y-axis and presented as a boxplot. Error bars show the mean ± standard error of the mean (SEM). Statistical significance was determined using a nested *t*-test (*$p < 0.05$**$p < 0.01$, ***$p < 0.001$, ****$p <$ 0.0001). **(C)** Quantitative analysis of Dcp2-EGFP puncta production per cell in wild-type and *nst1* deletion mutants. The total integrated density of Dcp2-EGFP condensates in each strain was determined. Error bars show the mean ± standard error of the mean (SEM). Statistical significance was determined using the Student's *t*-test *$p < 0.05$**$p < 0.01$, ***$p < 0.001$, ****$p < 0.0001$).

Fig S3

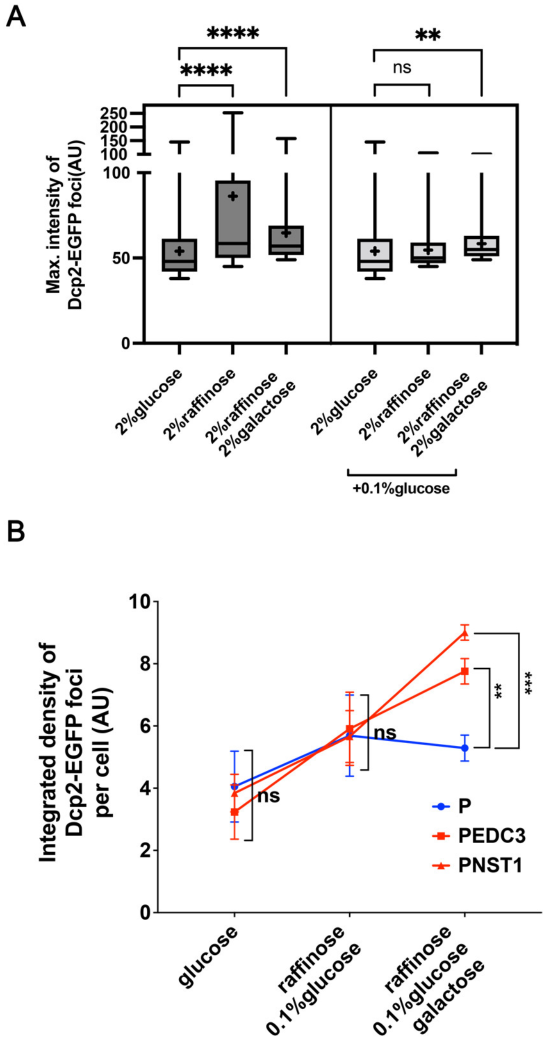

Figure S3. Monitoring the generation of Dcp2-EGFP puncta in different carbon sources to elicit the proper conditions for raffinose-galactose induction. (**A**) Wild-type cells expressing endogenous EGFP-tagged Dcp2 (YSK3458) were cultured exponentially in SD glucose medium (2% glucose) and collected to observe Dcp2-EGFP. These cells were transferred to 2% raffinose medium (without glucose) or 2% raffinose medium with 0.1% glucose for 3 h and collected to observe Dcp2-EGFP. The final concentration of 2% galactose was added to these cells to activate the *gal* promoter and further incubated for 3.5 h, and Dcp2-EGFP was observed in these cells. The cell number analyzed in each group was as follows: 23 for 2% glucose, 31 for 2% raffinose, 37 for 2% raffinose + 2% galactose, 32 for 2% raffinose + 0.1% glucose, and 49 for 2% raffinose + 2% galactose + 0.1% glucose. Pixels with the highest 0.05% Dcp2-EGFP signal intensity were segmented for the analysis. Segmentation and quantification methods applied at images are described in the Section 4. The maximal intensity of segmented puncta was plotted on the y-axis and presented using a boxplot. Error bar shows the mean ± standard error of the mean (SEM). Statistical significance was determined using a nested *t* test (*$p < 0.05$**$p < 0.01$, ***$p < 0.001$, ****$p < 0.0001$). (**B**) Nst1 and Edc3 were respectively overexpressed with 0.1% glucose-added raffinose-galactose induction condition in the wild-type cells expressing endogenous EGFP-tagged Dcp2 (YSK3458). In detail, exponentially growing cells in 2% glucose medium were transferred to 2% raffinose + 0.1% glucose medium for 3 h, and then 2% galactose was added, and cells were incubated for 3.5 h. The cells in each induction step were collected, and their Dcp2-EGFP was monitored. More than 150 cells of each step were analyzed for one independent experiment, and every experiment was repeated thrice. Total integrated density of Dcp2-EGFP condensates in Nst1 and Edc3-overexpressed cells was determined in each step and compared with that of the vector-only control. Error bar shows the mean ± standard error of the mean (SEM). Statistical significance was determined using a Student's *t* test (*$p < 0.05$**$p < 0.01$, ***$p < 0.001$, ****$p < 0.0001$).

Fig S4

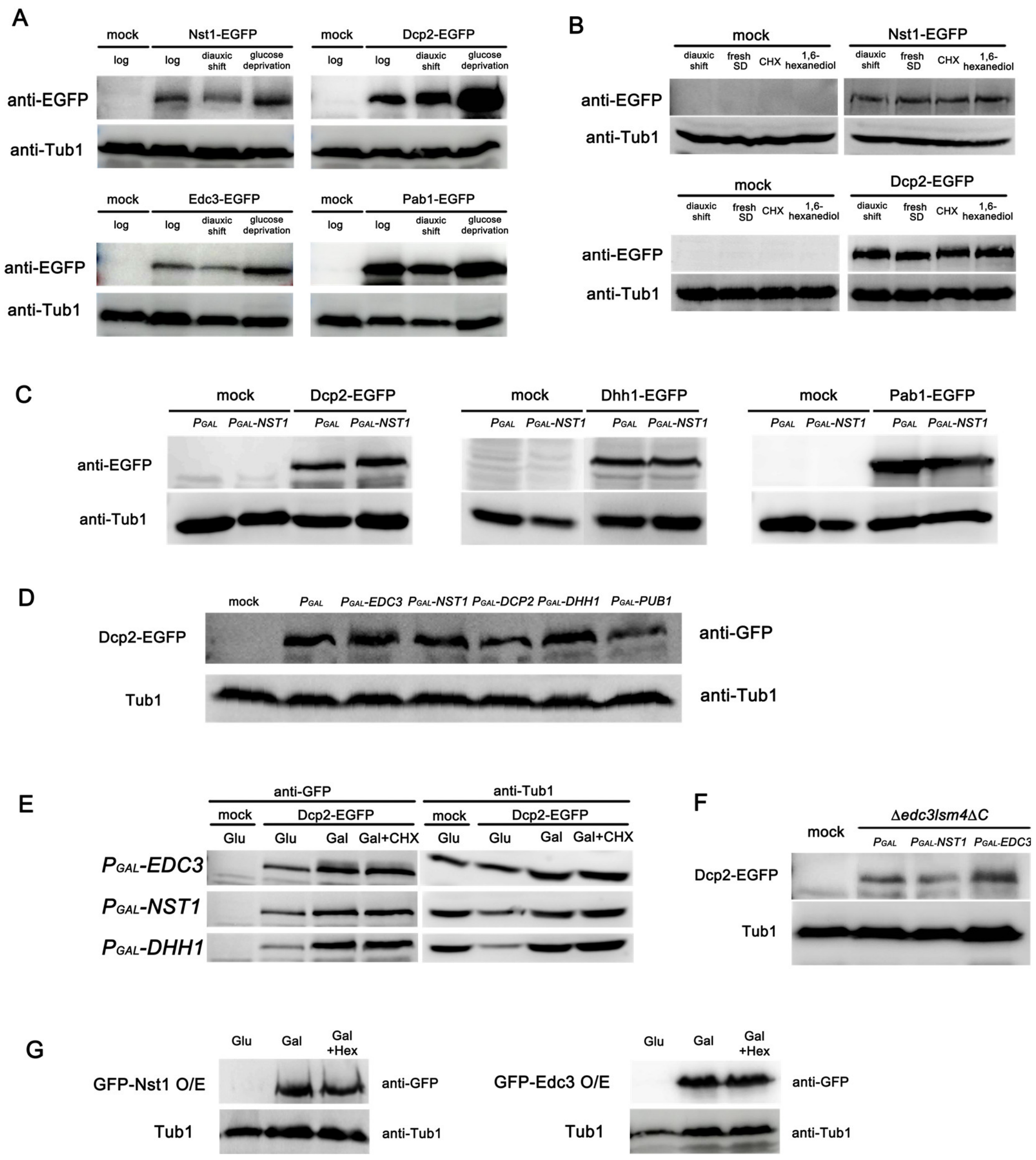

Figure S4. Expression levels of GFP-tagged proteins were confirmed using western blot analyses. The expression levels of Nst1-EGFP, Dcp2-EGFP, Dhh1-EGFP, and Pab1-EGFP were monitored using western blotting in each strain collected for fluorescence microscopy (Figures 1B, 1D, 2D, 3D, 4D, 5D, 6B). Anti-EGFP antibody (600-101-215 ROCKLAND) was used for western blotting, and the same blot was probed with anti-Tub1 (T5168, Sigma) as a positive control of expression. **(A)** Endogenous Nst1-EGFP, Dcp2-EGFP, and Pab1-EGFP expression levels in the log phase or stationary phase cells of Figure 1B. **(B)** Endogenous Nst1-EGFP or Dcp2-EGFP expression in each condition is shown in Figure 2A and B. **(C)** Endogenous expression levels of Dcp2-EGFP, Dhh1-EGFP, and Pab1-EGFP confirmed in the presence and absence of Nst1 overexpression as shown in Figure 3A. **(D)** Endogenous Dcp2-EGFP expression was confirmed in Nst1-, Edc3-, Dcp2-, Dhh1-, and Pub1-overexpressed cells in Figure 4A. **(E)** Endogenous Dcp2-EGFP expression during Nst1, Edc3, and Dhh1 overexpression by the *gal*-induction process and after treatment with cycloheximide (CHX) as in Figure 5A. **(F)** Endogenous Dcp2-EGFP expression levels in *edc3lsm4c* mutant cells in Figure 6A in the presence or absence of Nst1 overexpression. (**G**) Monitoring the overexpression of GFP-tagged Nst1 and Edc3 in Figure 7A, during *gal* induction and 1,6-hexanediol treatment.
